# Angiotensin in ECMO patients with refractory shock

**DOI:** 10.1186/s13054-018-2225-4

**Published:** 2018-11-01

**Authors:** Marlies Ostermann, David W. Boldt, Michael D. Harper, George W. Lim, Kyle Gunnerson

**Affiliations:** 1 Department of Critical Care, King’s College London, Guy’s & St Thomas’ Foundation Hospital, London, SE1 7EH UK; 2 Department of Anesthesiology and Critical Care Medicine, UCLA Healthcare System, West Los Angeles, USA; 3 Integris Baptist Medical Center, Nazih Zuhdi Transplant Institute, Oklahoma City, USA; 4 Departments of Emergency Medicine, Anesthesiology and Internal Medicine, Michigan Center for Integrative Research in Critical Care, University of Michigan, Ann Arbor, MI USA

Refractory vasodilation and catecholamine resistance are common in septic shock. Changes in receptor signaling, excessive production of nitric oxide, and absolute or relative deficiencies of vasoactive hormones, including cortisol, vasopressin, and angiotensin II, play a role. Angiotensin II (Ang II) was previously available as a vasopressor but removed from the market in the 1990s. Interest was re-ignited following the Angiotensin II for the Treatment of Vasodilatory Shock (ATHOS-3) study, a randomized controlled trial in patients with refractory shock which confirmed that Ang II was effective at maintaining mean arterial pressure and reducing norepinephrine requirements without an increase in side effects [1]. Patients receiving renal replacement therapy also had improved survival and faster recovery of renal function [2]. Recent literature noted the potential role of Ang II in other types of shock [3].

The major physiological effects of Ang II relate to maintenance of hemodynamic stability and fluid and electrolyte regulation (Table 1). Angiotensinogen, the precursor of angiotensin, is produced primarily by the liver and released into the systemic circulation where it is converted to angiotensin I (Ang I). Ang I is cleaved into Ang II, predominantly by angiotensin converting enzyme (ACE), an endothelium bound protein that is primarily expressed in the pulmonary and renal capillary beds. In patients with acute respiratory distress syndrome, ACE insufficiency has been reported [4]. In veno-arterial ECMO, a proportion of blood bypasses the lungs, which further limits the conversion of Ang I to Ang II. Other conditions associated with reduced Ang II levels include Gram-negative sepsis where endotoxinemia can deactivate ACE. Importantly, low levels of Ang II and ACE are associated with increased mortality [5].

**Table 1:** Main physiological effects of angiotensin II

Organ system	Physiological effects
Vascular	● Vasoconstriction of venous and arterial vessels ● Increased vascular permeability
Renal	● Stimulation of Na reabsorption and H^+^ excretion in the proximal tubule via Na^+^/H^+^ exchanger ● Stimulation of the release of aldosterone ● Variable effects on glomerular filtration and renal blood flow depending on the physiological and pharmacological setting: ➢ constriction of the afferent and efferent glomerular arterioles with greater effect on the efferent vessel ➢ constriction of the glomerular mesangium ➢ enhanced sensitivity to tubulo-glomerular feedback ➢ increased local release of prostaglandins which antagonize renal vasoconstriction
Endocrine	● Stimulation of the secretion of vasopressin from the posterior pituitary gland ● Secretion of ACTH ● Enhanced release of noradrenaline from postganglionic sympathetic fibers
Nervous	● Enhancement of noradrenaline secretion
Cardiac	● Mediation of cardiac remodeling through activated tissue RAS in cardiac myocytes
Coagulation	● Prothrombotic potential
Immune	● Promotion of cell growth and inflammation ● Increased expression of endothelium-derived adhesion molecules ● Synthesis of pro-inflammatory cytokines and chemokines ● Generation of reactive oxygen species

*Abbreviations*: *ACTH* adrenocorticotropin hormone, *Ang II* angiotensin II, *GFR* glomerular filtration rate, *RAS* renin-angiotensin system

We report the successful management of seven patients (four male; mean age 36 years) with severe cardiorespiratory failure and refractory shock treated with extracorporeal membrane oxygenation (ECMO) who received Ang II in the context of the ATHOS-3 trial [1] or a compassionate use program (Table 2). Following initiation of Ang II, a profound effect on blood pressure was seen and the doses of vasopressors were reduced quickly. Time to cessation of vasopressors and catecholamines ranged from 16 h to 8 days. Six patients were discharged home alive.

**Table 2:** Patient characteristics

	Patient 1	Patient 2	Patient 3	Patient 4	Patient 5	Patient 6	Patient 7
Age (years)	23	26	41	48	38	50	37
Gender	M	M	F	F	M	F	M
Primary acute illness	Influenza A infection	Sepsis	Influenza B and MRSA pneumonia	Sepsis post acute MI	Aspiration pneumonia	Pulmonary embolism	Type A aortic dissection
Secondary acute illness	Cardiac arrest due to pericardial effusion	Cardiac arrest	Sepsis and cardiogenic shock		Drug overdose (calcium channel blocker and beta blocker)	Multi-organ failure	Poly-microbial sepsis
Confounding factors	None	Idiopathic dysautonomy and mast cell activation syndrome	Obesity	HIV positive	Obesity	Recent craniotomy for meningioma	Large RV and LV infarct
Type of ECMO	VA ECMO	VA ECMO	VA ECMO	VV ECMO	VV ECMO	VA ECMO	VA ECMO
Vasopressor support *pre-Ang II administration	Norepinephrine 0.4 Vasopressin 4 Epinephrine 0.07	Norepinephrine 1 Vasopressin 6 Epinephrine 0.3	Epinephrine 0.18 Vasopressin 2	Norepinephrine 0.59	Norepinephrine 1.36 Vasopressin 2.4	Norepinephrine 0.2 Vasopressin 5 Milrinone 0.25 Epinephrine 0.05	Norepinephrine 0.1 Vasopressin 4 Epinephrine 0.02
MAP at initiation of Ang II [mmHg]	Missing	57	76	70	63	59	59
Dose of Ang II [ng/kg/min]	Missing	Missing	20	20	40	20	20
Duration of Ang II			7 days	46 h	50 h	27.5 h	80 h
Time to cessation of all vasopressors after initiation of Ang II	Missing	48 h	Missing	16 h	6 days	8 days	NA
Adverse events during Ang II infusion	None	None	Reversible digital ischemia	None	None	None	Bowel ischemia
Patient outcome	Survival	Survival	Survival	Survival	Survival	Survival	Deceased
Duration on ECMO [days]	17	5	119	4	9	9	14
Length of stay in ICU [days]	176	30	128	21	22	13	14

Abbreviations: *Ang II* angiotensin II, *ECMO* extracorporeal membrane oxygenation, *ICU* intensive care unit, *LV* left ventricle, *MAP* mean arterial pressure, *MRSA* methicillin-resistant *staphylococcus aureus*, *RV* right ventricle, *VA* veno-arterial, *VV* veno-venous

*Units of drugs: norepinephrine in μg/kg/min; epinephrine in μg/kg/min; vasopressin in units/h; milrinone in μg/kg/min

In conclusion, in patients with severe cardio-respiratory failure requiring ECMO, treatment with Ang II in addition to standard supportive care enabled rapid decatecholaminization. Underlying ACE deficiency may be a contributing factor. Further studies are necessary to confirm the findings.

## Abbreviations

ACE: Angiotensin converting enzyme
Ang I: Angiotensin I
Ang II: Angiotensin II
ATHOS: Angiotensin II for the Treatment of Vasodilatory Shock
ECMO: Extracorporeal membrane oxygenation
